# Biological Processes for Pollution Control: Current Research and Emerging Technologies 2020

**DOI:** 10.1155/2021/9852531

**Published:** 2021-11-05

**Authors:** Jin Li, Yu Tao, Guangbin Li, Cuijie Feng, Rong Chen, Ming Hua

**Affiliations:** ^1^Qingdao University, Qingdao, China; ^2^Chinese Academy of Sciences, Beijing, China; ^3^University of Maryland, College Park, USA; ^4^Polytechnic University of Milan Piazza Leonardo da Vinci, Milan, Italy; ^5^Xi'an University of Architecture and Technology, Xi'an, China; ^6^Nanjing University, Nanjing, China

With rapid economic growth, aggravated environmental pollution has become a severe issue worldwide [1, 2]. Pollution control and nutrient recovery are imperative for sustainable development and critical for ecosystem and human health [3]. Excessive chemicals and energy have been used to control pollutants, which causes neglected but severe side effects [4].

The biological process is regarded as promising and sustainable [5, 6], developing quickly with exciting breakthroughs in both theory improvement and technology innovation [7]. The bioparts of pollutants are degraded through microbial activities, where complex organic matters are degraded, and nutrients such as nitrogen and phosphorus are removed. Archaea and bacteria are essential in such processes [8, 9]. For example, anaerobic ammonia-oxidizing (anammox) bacteria are responsible for nitrogen removal from both engineered and natural water systems [10, 11].

Aside from pollutant removal, biological processes are integrated with energy production and resource recovery [12]. Archaea and bacteria play essential roles in converting carbon, nitrogen, phosphorus, and other pollutants into energy and valuable chemicals [13, 14]. However, process efficiency and stability issues remain unknown, such as microbiome dynamics, metabolic mechanisms, and identification of novel microorganisms. These investigations will optimize current biological processes and innovate emerging technologies.

Pollutant removal and nutrient recovery are two major themes in this special issue. C. He et al. presented a comprehensive insight into the feasibility and robustness of anaerobic ceramic membrane bioreactors for treating high-strength phenol wastewater [15]. D. Cui et al. investigated the adsorption capacity and mechanism of Pb^2+^, Cd^2+^, and Ni^2+^ by the extracellular polymeric substance (EPS) from *Agrobacterium tumefaciens* F2. EPS's potential as a bioadsorbent sheds light on the control of heavy metal pollution [16]. R. Yang et al. studied the response and adaptation of microbial communities to a completely autotrophic nitrogen removal over nitrite (CANON) system exposed to extreme alkaline shocks. This study gave implications for recovering CANON reactors after alkaline shock [17]. J. Xing et al. applied a microbial flocculant extracted from *Klebsiella pneumoniae* J1 to remove carbamazepine in wastewater and domestic sewage. This eco-friendly and highly efficient microbial flocculant is expected for practical applications in carbamazepine removal [18]. For nutrient recovery, X. Gu et al. applied anaerobic baffled reactors for biohydrogen production out of molasses wastewater and analyzed hydrogen-producing acetogen's performance under increasing organic loads [19]. X. Li et al. demonstrated the feasibility of cultivating marine macroalgae (*Chaetomorpha maxim*) in a moving bed bioreactor to remove nitrogen and phosphorus in aquaculture wastewater and produce macroalgae biomass, supplying an effective option to benefit aquaculture systems [20]. A. Sasi et al. developed a *Bacillus licheniformis* mutant strain that could produce hyperchitinase, which has the excellent potential of recycling and processing chitin waste from crustaceans and shrimp, adding value to the crustacean industry [21]. L. Su et al. applied thermophilic solid-state anaerobic digestion of agricultural wastes, i.e., corn straw, cattle manure, and vegetable waste, and investigated the effects of temperature, total solid content, and C/N ratio [22].

The novel design of techniques is also addressed in the special issue. Y. Liu et al. developed a highly sensitive and rapid method for measuring BOD by establishing a correlation between current and dissolved oxygen. Such a rapid BOD current sensing biosensor method is expected to be used for wastewater monitoring [23]. X. Cui et al. developed a novel tubular photobioreactor with improved potential for microalgal cultivation. The new design can provide a more suitable microenvironment for microalgal cultivation and increase microalgae yield [24].

Given all these advances in biological processes for pollution control and nutrient recovery, what does the future hold for us? The application of biological tools in solving environmental problems has a long history and is broad, covering many underpinning and allied technological areas. We hope that the ten articles that are included in this special issue would supply inspiration to researchers who work in both biological science and environmental engineering.

*Jin Li* *Yu Tao* *Guangbin Li* *Cuijie Feng* *Rong Chen* *Ming Hua*
